# Supplementary material for: The Impact of Post-COVID-19 Syndrome on Self-Reported Physical Activity
Source: Int J Environ Res Public Health. 2021 Jun 3;18(11):6017. doi: 10.3390/ijerph18116017 (PMC8199934; doi:10.3390/ijerph18116017)
Supplement: Supplementary file 1 [file ijerph-18-06017-s001.zip › ijerph-1226919-SI.pdf]

Supplementary material

<u>Summary of questionnaires</u>

Sports/activities:

- T0: Before the Corona infection I have performed the following sport or activities on a weekly basis.
- T1 and T2: During the past week I have performed the following sports or activities.
- Participants were asked to select all answer options that applied and we added an open text field in case the activity or sport was not mentioned in the answer options.

Walking:

- T0:
    - Before the Corona infection I walked **XX** times per week.
    - On average a walk/hike took **XX** minutes
- T1 and T2
    - During the past week I walked **XX** times
    - On average a walk/hike took **XX** minutes

Supplementary tables

Table S1. General characteristics of participants with suspected COVID-19.

	**Participants with suspected COVID-19 n=766**
**Women, n (%)**	652 (85.1)
**Age, years, median [IQR]**	48.0 [40.0-54.0]
**BMI, kg/m^2, median [IQR]**	24.7 [22.2-28.1]*
**Marital status – married/living with partner, n (%)**	543 (70.9)
**Education level, n(%)**	*
Low	19 (2.5)
Medium	258 (33.7)
High	484 (63.2)
Other	5 (0.7)
**Self-reported pre-existing comorbidities, n (%)**	
None	475 (62)
1 comorbidity	196 (25.6)
≥2 comorbidities	95 (12.4)
**Received care**	
**No care needed, %**	
Between infection and 3 months of follow up	9.5*
Between 3 and 6 months of follow up	13.4
**Physiotherapy, %**	
Between infection and 3 months of follow up	24.3*
Between 3 and 6 months of follow up	57.2#
**Rehabilitation, %**	
Between infection and 3 months of follow up	1.3*
Between 3 and 6 months of follow up	4.4#*

*p<0.05 vs. participants with confirmed diagnosis, #p<0.05 vs. 3 months of follow up.

Table S2. General characteristics of participants stratified into hospitalized and non-hospitalized.

	Hospitalized participants (n=62)	Non-hospitalized participants (n=177)
**Women, n (%)**	39 (62.9)	159 (89.8)*
**Age, years, median [IQR]**	53.0 [47.8-60.0]	48.0 [37.5-54.5]*
**BMI, kg/m^2, median [IQR]**	28.2 [24.8-32.6]	25.6 [23.0-29.4]*
**Marital status – married/living with partner, n (%)**	43 (69.4)	130 (73.4)
**Education level, n(%)**		
Low	6 (9.7)	0 (0)
Medium	32 (51.6)	94 (53.1)
High	24 (38.7)	83 (46.9)
**Self-reported pre-existing comorbidities, n (%)**		*
None	28 (45.2)	114 (64.4)
1 comorbidity	23 (37.1)	39 (22.0)
≥2 comorbidities	11 (17.7)	24 (13.6)
**Received care**		
**No care needed, %**		
Between infection and 3 months of follow up	4.8	18.1*
Between 3 and 6 months of follow up	12.9	14.7
**Physiotherapy, %**		
Between infection and 3 months of follow up	48.8	26.0*
Between 3 and 6 months of follow up	61.3	62.1#
**Rehabilitation, %**		
Between infection and 3 months of follow up	9.7	2.3*
Between 3 and 6 months of follow up	12.9	11.3#

*p<0.05 vs. hospitalized participants, #p<0.05 vs. 3 months of follow up.

Table S3. Activities performed by participants before (T0) and 3 (T1) and 6 months (T2) of follow up stratified into hospitalized and non-hospitalized.

	Sport/Activity, n(%)	Hospitalized (n=62)			Non-hospitalized (n=177)		
		T0	T1	T2	T0	T1	T2
1	Walking	28(45.2)	24(38.7)	41(66.1) *#	99(55.9)	75(42.4)*	122(68.9) *#
2	Cycling Outdoor	23(37.1)	17(27.4)	30(48.4) #	61(34.5)	34(19.2)*	71(40.1) #
3	(Physio)Fitness/Exercise groups	19(30.6)	7(11.3)*	29(46.8) #	53(29.9)	17(9.6) *	63(35.6) #
4	Swimming	5(8.1)	2(3.2)	1(1.6) *	19(10.7)	7(4.0) *	13(7.3)
5	Running	6(9.7)	2(3.2)	8(12.9)	18(10.2)	5(2.8) *	20(11.3) #
6	Yoga/Pilates	0(0.0)	0(0.0)	1(1.6)	12(6.8)~	0(0.0) *	6(3.4)
7	Racket sports	2(3.2)	1(1.6)	1(1.6)	8(4.5)	2(1.1) *	3(1.7)
8	Team sports	1(1.6)	0(0.0)	0(0.0)	7(4.0)	0(0.0) *	2(1.1)
9	Martial arts	1(1.6)	0(0.0)	0(0.0)	4(2.3)	1(0.6)	0(0.0)
	Cycling Indoor	2(3.2)	5(8.1)	7(11.3)	3(1.7)	5(2.8)	21(11.9) *#
	Work-related activities	2(3.2)	0(0.0)	0(0.0)	3(1.7)	0(0.0)	0(0.0)
10	Dancing	0(0.0)	0(0.0)	0(0)	4.(1.7)	0(0.0) *	0(0.0) #
	No sport/activities, regardless of COVID-19	7(11.3)	2(3.2)	1(1.6) *	20(11.3)	7(4.0) *	4(2.3) *
	No sports/activities, due to COVID-19	N.A.	24(38.7)	8(12.9)	N.A.	80(45.2)	20(11.3)

*: significant difference vs pre-COVID, p<0.05; #: significant difference vs 3 months, p<0.05, ~significant difference vs hospitalized, p<0.05.

Table S4. Walking duration stratified for sex.

	Male (n=41)	Female (n=198)	p-value
**Walking duration T0 (min./week)**	120[85-240]	120[60-225]	0.466
**Walking duration T1 (min./week)**	90[37.5-180]	50[10-105]	0.001
**Walking duration T2 (min./week)**	120[60-240]	80[30-142.5]	0.003
**Number of symptoms~**	11[9-15]	15[12-18]	0.000

~symptoms during the acute phase of the disease; Results are reported as median[IQR]; T0: before COVID-19; T1: after 3 months of follow up; T2 after 6 months of follow up

Table S5. Walking duration stratified for number of symptoms.

	Group A: 0 symptoms~ (n=0)	Group B: 1-5 symptoms~ (n=10)	Group C: 6-10 symptoms~ (n=41)	Group D: >10 symptoms~ (n=188)	Significant differences
**Walking duration T0 (min./week)**	-	90[90-165]	135[60-285]	120[60-221.25]	-
**Walking duration T1 (min./week)**	-	70[48-125]	120[60-195]	40[10-100]	Group C vs D: p=.000
**Walking duration T2 (min./week)**	-	97.5[30-162.5]	120[60-255]	77.5[32.75-140]	Group C vs D: p=.010

~symptoms during the acute phase of the disease; Results are reported as median[IQR]; T0: before COVID-19; T1: after 3 months of follow up; T2 after 6 months of follow up

Supplementary figures

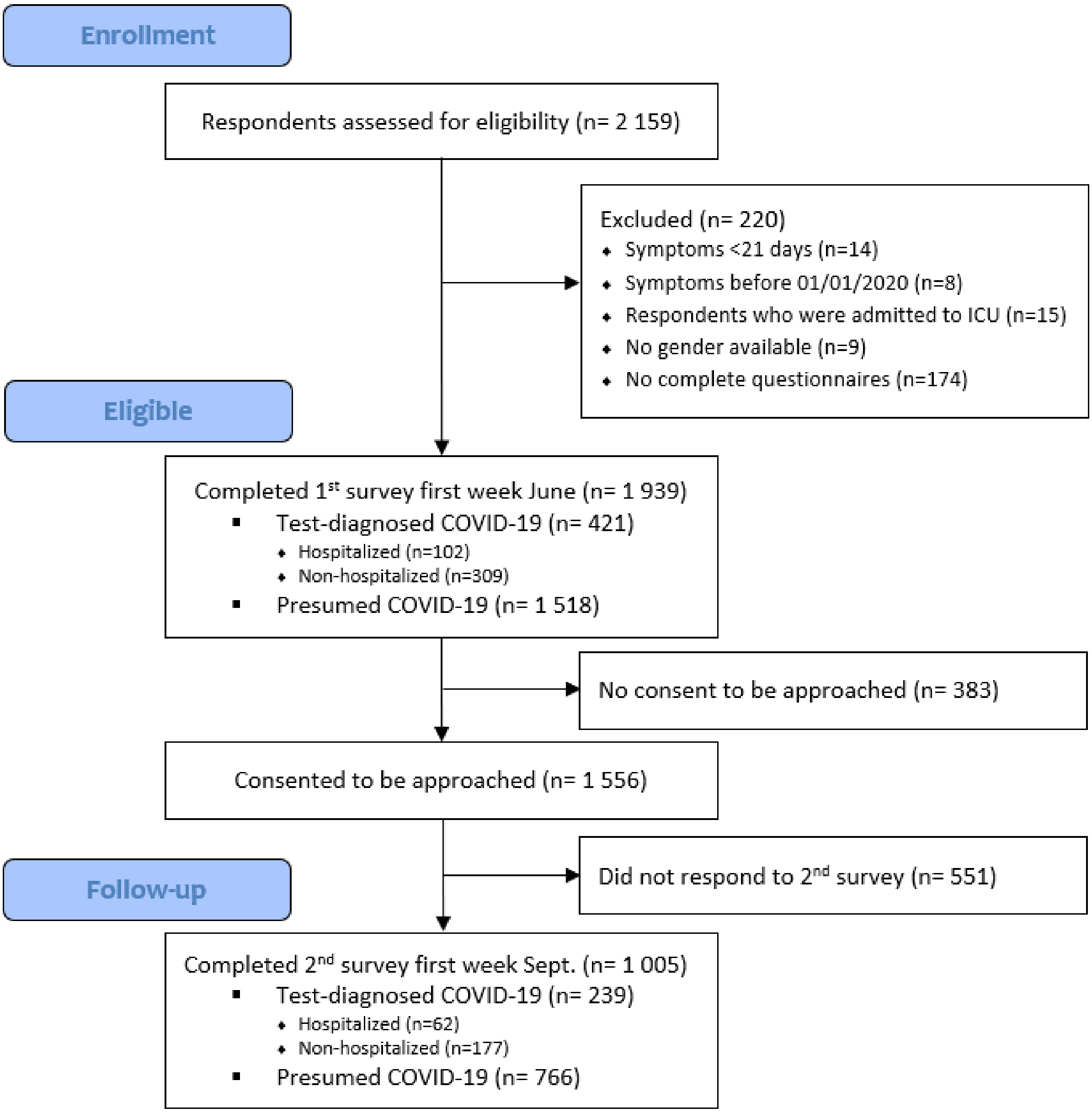

Figure S1. Flowchart of included and excluded participants.

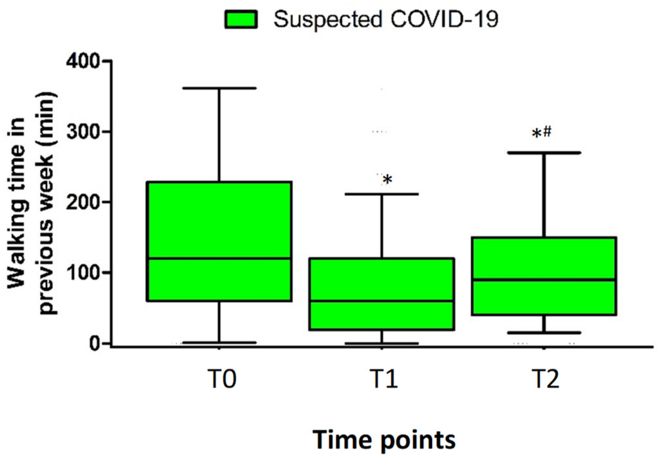

Figure S2. Walking time in previous week pre-COVID (T0), at 3 months of follow up (T1) and 6 months (T2) of follow up of participants with suspected COVID-19.

*: significant difference vs T0, p<0.05; #: significant difference vs T1, p<0.05; no significant differences vs patients with confirmed diagnosis. Data presented as median, IQR and 10-90%CI.

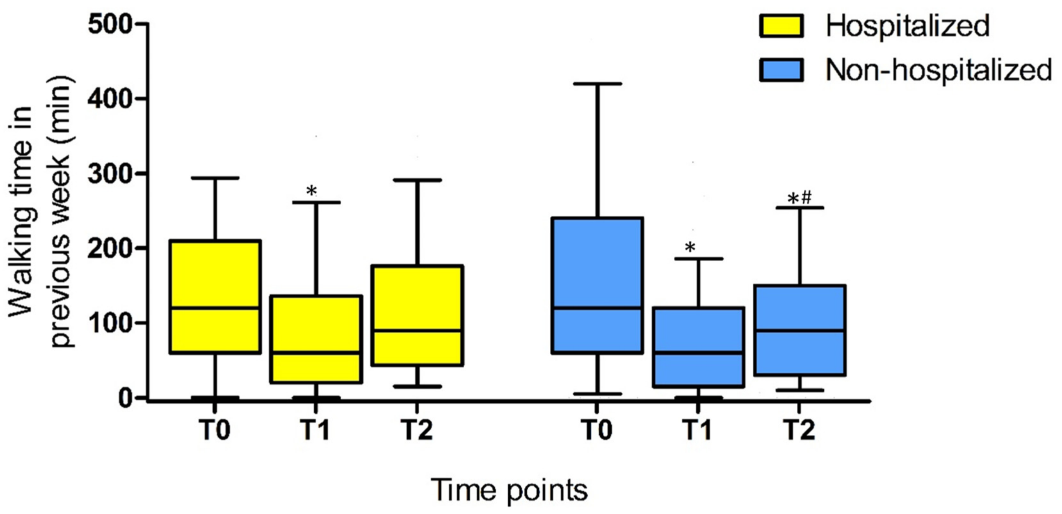

Figure S3. Walking time in previous week pre-COVID (T0), at 3 months (T1) and 6 months (T2) of follow up after stratification into hospitalized and non-hospitalized.

*: significant difference vs pre-COVID, p<0.05; #: significant difference vs 3 months, p<0.05

Data presented as median, IQR and 10-90%CI
